# Eco-Inefficiency Formula: A Method to Verify the Cost of the Economic, Environmental, and Social Impact of Waste in Food Services

**DOI:** 10.3390/foods10061369

**Published:** 2021-06-13

**Authors:** Maísa Lins, Renata Puppin Zandonadi, Virgílio José Strasburg, Eduardo Yoshio Nakano, Raquel Braz Assunção Botelho, António Raposo, Veronica Cortez Ginani

**Affiliations:** 1 Post-Graduate Program in Human Nutrition, University of Brasília, Brasília 70910-900, Brazil; 2 Department of Nutrition, University of Brasília, Brasília 70910-900, Brazil; renatapz@unb.br (R.P.Z.); raquelbotelho@unb.br (R.B.A.B.); vcginani@gmail.com (V.C.G.); 3 Department of Nutrition, Federal University of Rio Grande do Sul, Porto Alegre 90040-060, Brazil; virgilio_nut@ufrgs.br; 4 Department of Statistics, University of Brasília, Brasília 70910-900, Brazil; eynakano@gmail.com; 5 CBIOS (Research Center for Biosciences and Health Technologies), Universidade Lusófona de Humanidades e Tecnologias, Campo Grande 376, 1749-024 Lisboa, Portugal

**Keywords:** meal production, efficiency, food waste, sustainability metrics, waste prevention, food surplus, sustainability dimensions

## Abstract

This study aimed to develop an Eco-Inefficiency (Ely) formula to verify the cost of the economic, environmental, and social impact of waste, applicable to food services (FS). Six stages were performed: identification of the terms that characterize food waste; definition of constructs influenced by food waste; identification of the variables that make up each construct; indicators capable of measuring the impact generated by food waste; definition of the mathematical formula; and EIy pilot test. The formula was based on eco-efficiency but focused on food waste. The constructs were translated into three dimensions of sustainability: environmental, social, and economic. Researchers created a score for the dimensions and the entire evaluation, based on a literature review. Water footprint, cleaning material, food production waste, the amount of rest-intake, and the amount of distribution leftover were evaluated on the environmental impact. The economic dimension variables were energy consumption to produce the wasted food, cost of raw material used in wasted food, and food handlers’ wages for the economic impact measurement. The social impact variables were: energy density (ED), rest-intake (kcal/g), distribution of leftover ED (kcal/g), use of organic food, and food surpluses’ donation. With an EIy application in each item, we have the item’s score in each dimension. The higher value of an item, the higher is its influence on the dimension, allowing us to identify those with the most significant impact in the restaurant. The Environmental dimension presented the most significant problems in the assessed scenario. The eco-inefficiency formula identifies food waste’s main critical points, allowing us to trace strategies to reduce food waste.

## 1. Introduction

Food waste (FW) has become increasingly visible in academic debates and policies due to its detrimental impact on environmental, economic, and social issues [1]. Waste occurs at different points in the food production chain, and about one-third of produced food is discarded worldwide per year (46% occurring at the end of the food chain—meal production and distribution, such as in food services) [2]. Therefore, the investment added to food, such as energy and labor, increases the impact generated by waste and requires continuous control [3,4]. Food services (FS) must seek sustainable strategies to become environmentally, economically, and socially viable.

Food waste results from several events that demonstrate flaws in planning activities that involve meal production. It enhances the harm caused by these errors since the food in question is not used for its primary purpose and generates damage even when produced cleanly and consciously (i.e., using organic products, clean energy). It also demonstrates the need to analyze and identify the stages of the meal production process responsible for the losses and to adopt appropriate controls.

Sustainable food systems must consider the entire food production chain. The coherence between what is produced and how it is produced, as well as the destination given to this production, must be key points in the planning of each step that makes up the chain. In this sense, food services must propose menus that include foods that generate a minor impact on their production, but which also result in the least possible waste. For FW reduction to be a reality among different people, the United Nations proposed the 2030 Agenda [5]. 

The document consists of an action plan for governments, individuals, and companies in all countries with 17 sustainable development goals (SDGs) and 169 targets. Among the objectives is the encouragement of conscientious consumption and production of food, with goals of different nature. One of them is Goal 12, which proposes reducing FW at all stages of the food chain, and that the generation of waste is achieved through prevention, reduction, recycling, and reuse. The goal also observes the existence of public policies capable of supporting individual actions with impacts on the community, as in the case of food services [5,6]. 

However, the literature indicates that, for actions against waste to be effective, some gaps must be filled. Among the problems identified, the common point about FW is the absence of coverage required for the understanding and consequent transformation of the waste scenario. Estimates of waste, for example, are undersized, as is the impact caused by its generation. The partiality of studies concerning the object also implies results that are incapable of transforming reality. In this sense, the development of quantitative methods that seek to support interventions aimed at different aspects will help to solve such a serious problem that affects the entire world’s population [7,8].

A study used the idea of eco-efficiency (EE) (a set of actions that aims to use materials and energy more efficiently, reducing its impacts) to compare the range of environmental and economic impacts generated by meals from different menus or restaurants [9]. EE is usually calculated by the division of product value by environmental influence (EI) of production, often applied in large industries of different products [10,11,12,13]. Higher eco-efficiency values lead to smaller impacts, e.g., a menu/restaurant is more efficient if it has higher eco-efficiency scores. The findings are limited to the impacts of food produced, and there is no research focusing on meal production waste and its impact. However, the mentioned study makes it feasible to quantify aspects related to meal production that affect two sustainability dimensions. Beyond these aspects, there are social costs generated by food waste, such as hunger and a populations’ quality of life, as well as the fundamental values of buying and selling food [14].

Given the impact of restaurants on food waste production, it is essential to look for alternatives to identify and reduce overproduction and inappropriate food disposal. The opposite idea of eco-efficiency could be used to assess the impact of food waste, eco-inefficiency (EIy). Higher values result in more significant impacts on sustainability’s three dimensions. This study aimed to develop an Ely formula to verify the economic, environmental, and social impacts costs of waste generated by food services. From the production of meals’ data and the Ely formula application, each FS can evaluate their waste impacts and develop strategies to reduce waste, thus strengthening local sustainability.

## 2. Materials and Methods

**Figure 1:**
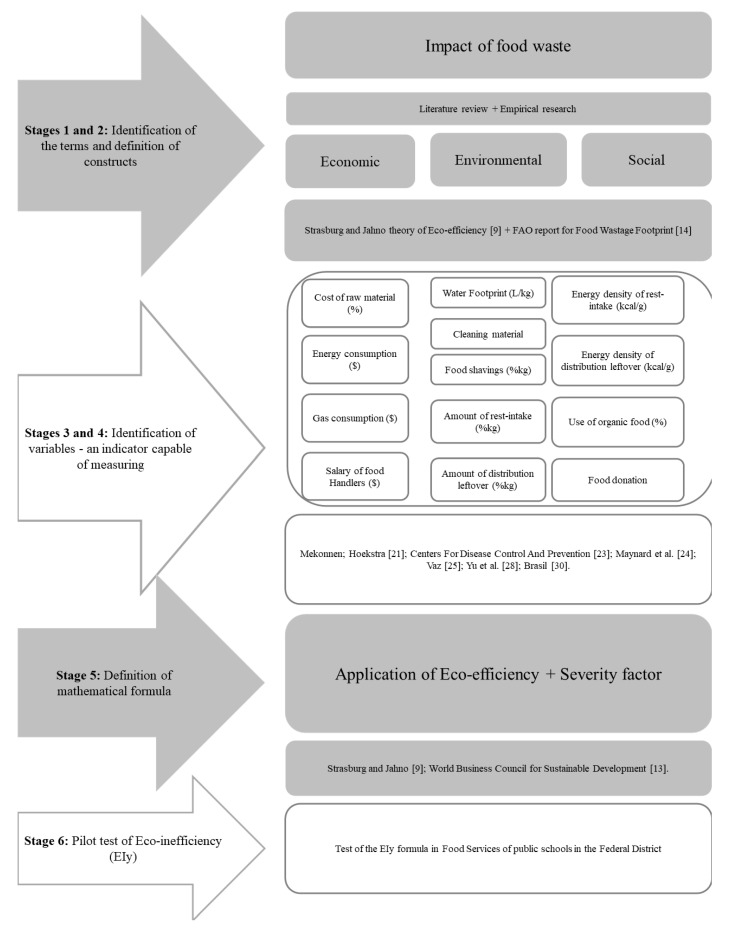
Diagram of the development of the mathematical formula of eco-inefficiency, a waste impact indicator on food services.

This cross-sectional study was developed in six stages (Figure 1). In this study, the term food waste refers to all food disposal in the final stages of the food supply chain, such as production, distribution, and consumption (food packaging waste was not considered) [15,16].

A diagram was created based on the variables belonging to each of the constructs (economic, environmental, and social) (Figure 1) and according to the protocol proposed by Sellitto and Ribeiro [17].

### 2.1. Identification of the Terms, Variables, and Definition of Constructs

Scientific literature was reviewed at stages 1 and 2 to identify existing tools to assess meal production’s impact on sustainability [9,14,15]. The items that composed the impact of food waste in FS were listed, described, and classified to determine numerical values. At stages 3 and 4, based on the literature and the researchers’ previous experience in FS, the information that would potentially compose the EIy formula was discussed to identify the variables related to each of the constructs. Researchers identified that one item could have more impact than others on food waste dimensions (social, economic, and/or environmental). However, by consensus, each item was allocated the dimension in which it was most influential in enabling the calculation of EIy.

### 2.2. Definition of Mathematical Formula

At stage 5, a mathematical formula was proposed to create an indicator capable of calculating the impact of the FS’s food waste. For that, the concept and the formula of eco-efficiency of the World Business Council for Sustainable Development (WBCSD) [13] were used:

(1) $$\mathrm{Eco}-\mathrm{efficiency}=\frac{\mathrm{Value}\;\mathrm{of}\;\mathrm{product}}{\mathrm{Environmental}\;\mathrm{influency}}$$

A study that applies the WBCSD term in FS to structure the eco-inefficiency formula was considered [9]. The term eco-inefficiency was used to approach all impacts generated by food waste, not by food production, as in eco-efficiency assessed by Strasburg and Jahno [9].

In the EIy formula, the severity factor (SF) was used in each item to measure its impact intensity. The SF was based on the criteria in the scientific literature or references to official documents related to the energy range, acceptable percentage of waste disposal, gas and electricity consumption range, adequacy in the use of cleaning material, food handlers’ salary range, water footprint value of the menu, energy density range of wasted food, use of organic food, and destination of food surplus. For each variable, a three-level interval of SF was determined.

The first level has the least impact on the construct, the second is intermediate, and the highest level is the classification with the greatest impact on food waste related to that item. The classifications were based on consolidated references described in the results of this work. The greater the SF, the higher the impact caused by the variable within that construct and, consequently, the more eco-inefficient the menu.

Thus, the mathematical EIy formula considered different weights within the same item through the SF, without ignoring the food wasted, which was the main reason for the formula.

### 2.3. A Pilot Test of the Eco-Inefficiency Formula

A cross-sectional study, previously approved by the Ethics and Research Committee of the University of Brasília (CAAE n° 02033218.0.0000.0030), was performed in public elementary schools in the Federal District, Brazil. Schools were raffled based on the list of schools participating in the School Health Program, serving just one daily meal to students.

For the pilot study, for five consecutive days, data for applying the formula during meal production were collected in each school using the data record sheets. A trained researcher collected data from four school FS, totaling 20 meal menus between March to June 2019 (Supplementary Material Table S1). The technical preparation files (TPF) (Supplementary Material Table S2) were used to register information collected about the produced meals. Therefore, during the pilot test, information was collected about all the necessary items for meals’ production and registered in the TPF: (1) weight and description of raw material; (2) cleaning material; (3) consumption of electrical energy; (4) liquefied petroleum gas consumption; (5) labor cost; (6) food shavings weight; (7) yield; (8) rest-intake; (9) distribution leftover; (10) destination of food surplus; (11) supplier.

All the ingredients were weighed on a digital scale (capacity of 5 kg and precision of 1 g) (MF Imports^®^, Brasilia, Brazil). For meals that exceeded the maximum weight of this equipment, we used a digital bench scale with a capacity of 300 kg that has an accuracy of 100 g (Toledo^®^, Brasilia, Brazil). Therefore, we recorded each ingredient’s weight before any type of manipulation, called gross weight (GW), and its respective net weight (NW), which is the weight after removing damaged or unfit parts for human consumption, also known as shavings. We also recorded the total weight of the ready-to-eat preparation, named yield preparation (YP) and the amount of wasted food (AWF). The proportional waste gross weight was calculated according to Equation (2).

(2) $$\mathrm{WGW}=\frac{\mathrm{AWF}\;\times \;\mathrm{GW}}{\mathrm{YP}}$$

where:

WGW—wasted gross weight.
AWF—amount of wasted food.
GW—gross weight.
YP—yield preparation.

When it is not possible to separate each food’s exact quantities that have been discarded, the disposal is considered proportionally to what was produced for composed meals. The costs of raw materials were obtained through the registration of auctions made available by the State Department of Education to purchase food. The percentage of wasted raw material cost was calculated according to Equation (3). The raw materials’ costs were obtained through the FS’s invoice records of raw materials acquired for food production.

(3) $$\%\;\cos t\;\mathrm{of}\;\mathrm{WRM}=\frac{\cos t\;\mathrm{of}\;\mathrm{WGW}}{\cos t\;\mathrm{of}\;\mathrm{GW}}\times 100$$

where:

WRM— wasted raw material.
WGW—wasted gross weight.
GW—gross weight.

Liquefied petroleum gas (LPG) and electricity usage were considered for energy consumption, so we divided them into these two categories. LPG consumption was estimated through the purchase frequency and replacement of the cylinder and its capacity (in m^3^). To obtain wasted LPG consumption, we made the proportion of gas consumed to produce the meal that was wasted (Equation (4)). Information on LPG consumption was obtained by observing the invoices (I) of last year’s purchase.

(4) $$\mathrm{wasted}\;\mathrm{LPG}=\frac{\mathrm{LPG}\;\mathrm{used}\;\mathrm{to}\;\mathrm{produce}\;\mathrm{the}\;\mathrm{meal}\;\times \;\mathrm{wasted}\;\mathrm{food}}{\mathrm{YP}}$$

where:

LPG—liquefied petroleum gas.
YP—yield preparation.

The local electricity company provides information on the stipulated price for specific monthly consumption ranges (low, medium, or high). These same ranges were used to classify electricity consumption in the restaurant’s daily proportion [18]. The consumed electric energy was gauged by recording the operating time of the equipment connected to the electrical network in the production of the meal. Additionally, the average consumption in Wh (Watts hour) or Kilowatt-hours (KWh) of each one supplied by the manufacturer was used to calculate the voltage consumed per hour (for each equipment) [19].

Concerning labor costs, we obtained information on employee expenses (salary, vacation, transportation) involved in meal production through data on payrolls. The water footprint (WF) value of the reference tables published by Hoekstra [20] and Mekonnen and Hoekstra [21] was multiplied by the GW of the food waste to obtain the WF of the disposal. The reference table value was multiplied by the equivalent GW of the wasted food to obtain the WF of waste.

Data from the TPF were used (ingredient weight, yield, and weight of wasted food—distribution leftovers and rest-intake), and the proportion of the waste gross weight was calculated. The WF of the waste was established by the sum of the wasted food’s WF considering the type and amount of the ingredients used to prepare all discarded dishes. Therefore, knowing each ingredient’s gross weight, the dish yield, and the food waste weight, we could obtain the waste gross weight (Equation (2)). The GW value of the waste of each ingredient identified in the dish is multiplied by its equivalent WF value, defined by Hoekstra and Mekonen [20,21], and the sum of these values corresponds to the WF of the wasted dish. The existence of Work Instructions (WI) or Standardized Operating Procedures (SOP) were used to verify the conformity of using cleaning materials, indicating the correct amount of dilution and whether the food handlers were following procedures correctly. In the absence of these documents, we directly observed the dilution and the use of these products. Any incorrect practice was classified as inappropriate, suitable only for those who followed WI’s guidelines, SOP, or the manufacturer to use any cleaning products.

The weight of the production leftovers (PL) and the weight of the produced food were recorded. The PL is considered suitable for reuse if the food is not displayed on the distribution counter, and it is stored under adequate and controlled time/temperature conditions. In the case of inadequate storage, PL is considered a distribution leftover (DL). The DL corresponds to the exposed/served food that cannot be reused for human consumption and needs to be discarded. The rest-intake (RI) is the remaining food on the customer’s plate [22]. To calculate the percentage of rest-intake (%RI) and the percentage of distribution leftover (%DL), we used the formulas described in Equations (5) and (6), respectively.

(5) $$\%\;\mathrm{RI}=\frac{\mathrm{WRI}}{\mathrm{YM}}\times 100$$

(6) $$\%\;\mathrm{DL}=\frac{\mathrm{WDL}}{\mathrm{YM}}\times 100$$

where:

RI—rest-intake.
WRI—wasted rest-intake.
DL—distribution leftover.
WDL—wasted distribution leftover.
YM—yield of the meal.

Through TPF, the energy density (ED) was determined by the amount of energy (kilocalories) per amount of food (grams) (Equation (7)). For the ED of RI, the RI energy value was divided by the RI weight [23]. The same calculation idea was used for the ED of the DL. Microsoft Excel platform (2007) was used to facilitate the formula’s application.

(7) $$\mathrm{ED}=\frac{\mathrm{EV}}{\mathrm{FW}}\times 100$$

where:

ED—energy density.
EV—energy value (kcal).
FW—food weight (g).

## 3. Results and Discussion

**Figure 2:**
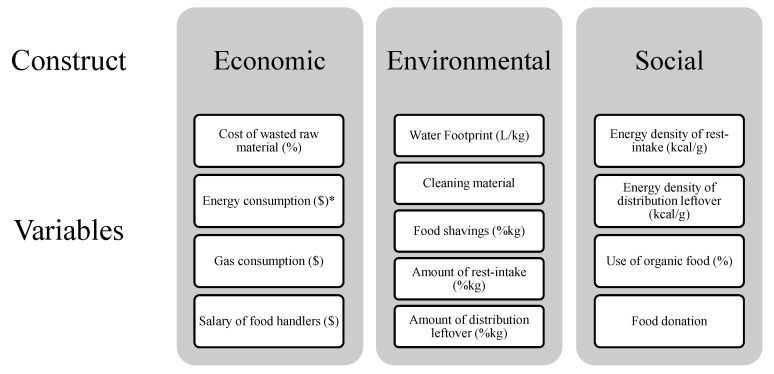
Aspects included and evaluated in each dimension (environmental, social, and economic) of the food waste impact. (*, This item considers all forms of energy consumption of the food service evaluated. This item will be divided later, depending on the food service. In the case of exclusive use of electricity, only one item should be considered; in food services that use electricity and liquefied petroleum gas, for example, this item will be stratified into two parts (as used in this study).

The formula and concept of eco-efficiency proposed by Strasburg and Jahno [9], FAO’s [14] considerations on food waste, and the eco-efficiency theory of WBCSD [13] were used as the primary basis for the elaboration of the EIy formula. Figure 2 shows all the items included and evaluated in each sustainability dimension related to food waste.

### 3.1. EIy Formula

The EIy formula (Equation (8)) was defined to obtain a standardized score of the waste’s exclusively generated impact for each evaluated item. Even though an item can impact more than one dimension [24], we adopted each item’s use in a single sustainability dimension (Figure 1). Although the economic and environmental dimensions contain a higher number of items than the social one, they are not considered more relevant. More items fit better in these economic and environmental dimensions [24].

(8) $$\mathrm{EIy}={\left(\frac{\mathrm{Food}\;\mathrm{wasted}\;\left(\mathrm{kg}\right)}{\mathrm{Meal}\;\mathrm{produced}\;\left(\mathrm{kg}\right)}\right)}^{\frac{1}{\mathrm{Severity}\;\mathrm{factor}}}$$

The SF classifications used in the EIy formula are described in Table 1. The SF values were obtained by combining the proportion of waste (ratio between the amount of food wasted and the total amount of food produced) power to an SF.

The formula is applied to each item that comprises eco-inefficiency (Figure 1 and Figure 2). The SF determines the intensity of the impact generated by each item, assessed at the FS. Each evaluated item is graded in degrees of intensity (from 1 to 3). For example, if two restaurants generate the same wasted food/meal produced ratio, the SF can differentiate them according to how the food is produced.

The calculations made in the pilot study are presented in the Supplementary Material (Tables S3 and S4); the first with the base calculation of the wasted food/meal produced ratio and the second with the database and the corresponding SF values for each of the formula’s items. For a restaurant evaluating their menus, the most eco-inefficient menu can be replaced, or the most eco-inefficient dishes can be changed for other dishes, thus reducing the restaurant’s waste.

These items were classified and described in Table 1 according to the level of impact they can have on the environment (SF), with the first level being the least impact and level 3 being the maximum impact. The higher the SF, the more significant the item’s composition’s impact, resulting in a higher score. It is worth noting that a menu evaluated by the eco-efficiency methodology as eco-efficient can be eco-inefficient at the same time, in case it does not have good acceptance or presents problems in the management of resources that favor waste. Therefore, EIy’s evaluation shows itself as a possibility for a broader analysis that is important to reduce the meal production system’s impact [9].

The application of the formula results in a value between 0 and 1. The eco-inefficiency value can be assessed by dimension or the sum of the three dimensions in each restaurant. Each dimension´s score is obtained by the sum of their items’ scores, and the total score is the sum of the points of all dimensions.

Therefore, each sustainable dimension’s maximum value is associated with the number of the defined items shown in Table 1. For the environmental impact, the maximum score is 5; for the economic impact, the maximum is 4 points; and for the social dimension, the maximum is 4. If a restaurant scores maximum points, the total score will be 13 points. All of the items are important and must be considered for the formula.

In terms of the absence or impossibility of measuring the data referring to one or more items in (Table 1), the worst possible value should be considered (3). Higher scores are associated with high EIy. This value will only be zero when the produced waste ratio and the food produced is zero, e.g., when there is no waste in the evaluated menu.

#### Defined Parameters for the Application of EIy Formula

A clear understanding of the net economic benefits associated with each item, and its associated environmental and social effects, increases transparency and could create incentives for reducing food waste [31]. The database for each item was obtained from the literature review within the production area.

The cost of the wasted raw material (WRM) is essential because it reveals the discarded food’s financial cost, estimated at almost USD 750 billion per year [2]. This economic value of purchasing raw materials is most often perceived. Every restaurant manager must adhere to this aspect to control food waste and financial losses [2].

Regarding the amount of wasted gas, the reference intervals were defined based on a Brazilian company’s information (Copergás) [26] related to charging for gas consumption in m^3^/day multiplied by the same company’s price.

The average monthly salary of food handlers directly involved in meal production (the hours worked in the wasted food production) was included. This value has a significant impact on the FS’s economy since all training carried out with the handlers generates expenses, in addition to wages and wasted working hours [32]. In the EIy formula, the proportion wasted from all employees’ daily wages involved in the meal production was considered. This calculation considered the relationship between the paid wage for total food production and what was wasted. Therefore, it estimates how much is discarded in production.

Salary levels were adjusted to adapt to what is used by the Brazilian Institute of Geography and Statistics (IBGE) [27]; five levels are reported in monthly values. This value was converted into daily ranges. Initially, there were seven (below USD 11.80 per day; USD 11.81 to USD 17.70; USD 17.71 to USD 35.39; USD 35.40 to USD 58.98; USD 58.99 to USD 88.48; USD 88.49 to USD 147.46; and above USD 147.47—considering conversion rate of BRL 5.39/USD). The adapted version brought together the three lowest levels and the three intermediate levels, keeping the last classification equal, totaling three classifications represented in Table 1 [27]. This value was inserted in EIy, as it represents the amount spent on the payment of a restaurant employee along with food waste.

To calculate environmental impact, Strasburg and Jahno [9] considered the water footprint (WF) of the food. By definition, WF is all water volume used directly or indirectly in food production. The WF indicator is one of the primary assessments of water consumption and one of the most significant environmental impacts caused by food production [33].

The definition of the WF interval ranges was determined after the pilot data. The water footprint values were distributed in terciles based on the cutoff points after calculating the produced food’s WF. Thus, the classification by category was established considering: Category 1, the evaluated establishments with the lowest WF (<33%); Category 2, between 33% and 66%; and Category 3, those with the highest WF (>66%).

Studies have pointed out some limitations of the WF as an indicator of sustainability. It does not consider factors such as climate change or social and economic aspects. For this reason, we considered other environmental sustainability indicators for application in the EIy formula [20,21,34,35,36].

For cleaning materials, the improper use of these products can cause water pollution due to incorrect dilution and foodborne diseases [37]. Therefore, regardless of the wasted food, cleaning material is considered wasted when it is misused because, in this case, the use of this product is inefficient and potentially generates waste. In this sense, this classification is limited to the correct or incorrect use; therefore, it is classified in only two extreme SF points: the most adequate (1) or the worst classification (3).

Chemicals are sometimes discarded, and they end up in streams and rivers without the correct treatment. Some do not disintegrate and enter the food chain. Packaging can also be a polluter when garbage is not adequately controlled and separated. This type of control is needed and should be encouraged by public policies and initiatives for selective garbage collection [37,38].

According to Papargyropoulou et al. [39], employees are often not careful in restaurants with higher food production while cutting or peeling food. They do not receive adequate training in avoidable waste parts of the food. Another reason is the absence of processes that facilitate food handling, or the overwork that can generate fatigue and a lack of commitment [39,40].

The quantity of food wasted (amount of rest-intake and distribution leftover) must also be considered an environmental impact, highlighting this waste’s destination. The consumption of carbon and heavy metals generated by the garbage in landfills can affect the human ecosystem up to 100 years after the landfill’s extinction. FAO [2] estimated that, if food waste were considered a country in terms of greenhouse gas emissions, it would be the third-largest generator in the world [2,41].

For these items, we used the recommendation proposed by Vaz [25] that considers acceptable values of rest-intake below 3% (Table 1). Many studies used that same classification of rest-intake and leftover food waste [25,42,43,44,45,46].

Beyond the environmental and economic effects of food wastage, there may also be social effects. The social dimension of sustainability refers to the loss of well-being and quality of life suffered by humans [14]. This dimension is also directly related to the human right to adequate and healthy food [47].

According to the guidelines for social life cycle assessment of products, several factors are related to the social dimensions of food waste [48]. In order to apply the EIy formula, all the items that could be measured in food services that could reflect the reality of the impact of food waste in the social dimension were evaluated.

In this sense, for the calculation of EIy, we also inserted the energy values of wasted meals as a social factor through the energy density (ED) of the dishes [9,49]. We inserted the ED, because in the FAO publication, the social dimension must also be considered as one of the several factors directly or indirectly impacted by waste, generating costs [14].

In the present study, we grouped the “very low” and “low” classifications of ED reference established by the Centers for Disease Control and Prevention (CDC) to maintain only three intervals [23].

Therefore, the social aspect must also be included since discarded food is inaccessible and food waste can be directly related to hunger. The use of a nutritional parameter to measure food waste is a differential applied in few studies. The use of ED value suggests including a relevant and general social aspect suitable for any community [9,31]. Inserting information about micronutrients is also very important for the work and could undoubtedly enrich the formula, but it would be necessary to observe specific aspects of the studied population, considering different realities concerning existing nutritional deficiencies.

Menu planning, the meals’ acceptance, and the dishes’ presentation are essential strategies to reduce waste. They also make paid salaries in the restaurant more efficient and profitable [24,50,51]. Additionally, the disposal of food can represent emotional discomfort to those who produce it. Often (especially in developing countries), a food handler is a low-income individual and may experience the lack of this discarded food at home on a daily basis [52].

As a social domain, food donation is often suggested to reduce waste [24,53,54]. The literature considers redistribution of food waste to food charities (results in several meals given to people), as well as the number of meals saved and subsequently donated. However, it is also perceived as a concern to restaurant owners. Legislation may prohibit this type of destination. Additionally, there is a responsibility for the food’s safety [55].

Since, during the pilot study, restaurants were not allowed to donate food surplus during the period in which they were evaluated, they all had the highest score in that category. The law that allows food donation in Brazil was later released (June 2020) [30]. Therefore, this waste could not be considered actual waste. Researchers decided to insert this item in the formula considering the importance of this destination in reducing hunger.

However, a study shows that FS are still very concerned about potential punishments when donating [56]. Several initiatives in the country promote more rational use of food, such as Save Food Brasil, in partnership with the WHO, the #SemDesperdício (#NoWaste) initiative in partnership with FAO, and Embrapa [57,58].

All of these initiatives already existed in the country, even before the legislation approval encouraging food donation. Another possibility is to use surplus food for animal feed or composting [59]. The application of EIy can help restaurant owners and managers use these opportunities for food destinations, thus reducing the impact generated by them.

EIy also highlights the importance of public policies that encourage sustainable actions in restaurants and companies. The absence of these investments may be related to the scarcity of research on the subject in countries such as Brazil [24].

The consumption of organic products is greatly strengthened and encouraged by society and by political initiatives. Government support and inspection of pesticides’ use are essential for reducing the number of people affected [60].

Consuming organic foods instead of those with pesticides presents positive results in those who consume these foods, as better cognitive development in children demonstrates [61]. Besides, most organic producers sell their products directly to the consumer, reducing the impacts and losses resulting from transportation, as well as being extremely important for the trade and production of a given region [61]. Therefore, another way to assess the social influence of food waste is by analyzing the percentage of pesticides used in the items that make up the menu through organic products, an important sustainability indicator. Restaurants are considered sustainable when more than 50% of the menu’s fruits and vegetables have organic certification [24]. Therefore, when applying the EIy formula, only two levels of severity are considered: the lowest and the highest (1 and 3, respectively). This criterion occurs because the use of over 50% organic fruits and vegetables is considered adequate, scoring 1 in the SF; however, places where less than half of the fruits and vegetables are of a certified organic origin score 3 points.

### 3.2. Pilot Study

In Brazil, almost USD 450,000 is allocated to serve food to more than 40 million students from public schools throughout Brazil [62]. These data demonstrate the importance of public spending on food and the impact that waste can generate in restaurants, as they are places where waste monitoring must be a reality. In this sense, the proposed EIy was tested on the menus of Brazilian (Federal District) public schools’ restaurants. Table 2 presents the results of the sustainability dimensions of food waste.

The day with the highest total EIy score was also the day with the highest value of the ratio between the amount of food wasted/amount of food produced.

With the formula’s application to each item, we have the item’s scores in each dimension. The higher the value of an item, the higher the influence of this item on the corresponding dimension. Therefore, it is possible to identify those with the most significant impact on the restaurants’ food waste.

With the wasted value standardization (quantity discarded/quantity produced), the score directly expresses each item’s impact on the environment, the economy, and society. It is noteworthy that the higher the proportion of waste regarding production, the more eco-inefficient a restaurant will be, with a different intensity according to each item’s score.

It is evident that the primary need of the SF is to reduce the amount of waste since, the higher the food wasted/food produced ratio, the higher the score of this establishment/restaurant. However, the indicators presented in the “EIy formula” enable a targeted approach to minimize impacts as much as possible. It can be seen in the results of the pilot study that the environmental dimension is the one that demands the most attention when evaluating the percentage of points reached by each dimension. The higher the percentage, the greater the influence of that dimension on the restaurant’s eco-inefficiency, showing that it is necessary to pay attention to the items.

For example, the environmental dimension score, with 74.60% of the maximum score, highlights the need to provide awareness considering correct consumption to minimize the return of food and, therefore, high waste. This fact is evident when we analyze the proportion and identify the dimension that is not as adequate as the others (Table 2).

These numbers show that, in terms of the average of the establishments evaluated, the items that make up the environmental dimension must have more rigorously controlled and targeted strategies to minimize the impacts of this dimension. For example, in the cases studied where %rest-intake and %distribution leftover were high, a possible strategy is food and nutrition education with children highlighting the importance of conscious consumption, as well as campaigns and games that encourage a reduction in rest -intake [63].

In addition, food handlers also need to receive training in manipulation and good food hygiene practices in order to correctly portion the food (if served by them), to decrease rest-intake. Another important topic to instruct handlers is proper storage of ready-to-eat food, as the food kept in the proper condition of time and temperature can be distributed in food donations with total safety to whoever is going to receive it [30,63].

Once again, it can be seen that the dimensions interrelate and the assessment using the eco-inefficiency formula enables targeted actions, offering more immediate results.

We can also see that the scores obtained by applying the formula in each institution generated different indices that can be used to compare different establishments; these scores could be a motivating index for improvements to be made and a potential parameter to qualify the more sustainable restaurant. In the pilot study, we can classify schools by their level of efficiency, with school 3 (total score of 7.11) being the most efficient, followed by school 4 (with 7.68 points), then school 1 (8.63 points), and finally school 2 (a score of 8.88).

This comparison between establishments can encourage measures that reduce impacts, such as the quality seal for the food sector made in Brazil to improve food quality during the 2014 World Cup, hosted in the country, which classified food services as A, B or C. The better the classification, the more tourists would be attracted to the establishment, motivating managers to comply with the required parameters and providing higher quality food [64].

It is also worth mentioning that society’s concern with sustainable production is increasing, so it is of great value for an establishment to be highlighted as a “sustainable establishment” [65].

A more sustainable school campaign could also be developed on an annual basis. Considering the pilot study in this campaign, school number 3 would be the “champion”, motivating this establishment to remain at the top and seeking evidence to reduce its impacts and demanding assertive measures from other institutions for better results in the next edition of the campaign. Despite using a convenience sample, the pilot test’s primary purpose was to identify the formula’s efficiency. The formula enabled the identifying of the items with the most significant impact on the wastage cost in FS. Additionally, items could be compared, and the FS could adjust critical points. In Brazil, almost USD 8.08 million are used to buy food for more than 40 million students in public schools [62]. These data demonstrate the importance of public spending on food for students.

The results showed that the dimension with the most significant problems was the environmental one because the score was closer to the maximum value. These pilot findings show that the formula makes it possible to identify, within a restaurant, the critical points of production and to make comparisons between establishments.

In Brazilian public schools, it is relevant to analyze children’s menu and their acceptance thereof. Portion size also influences the wasted amount, and employees who participate in portioning should be trained to serve less food and allow repetition. In the case of self-service restaurants, it is crucial to make consumers aware of their serving. They should put on their plates only what they will eat and repeat when necessary. One option to reduce the rest on the plates is to present incentives for those who do not leave leftovers [24].

The evaluated schools’ FS had a similar economic dimension pattern because they are inserted in a centralized distribution system in the Federal District. The schools receive food and cleaning materials from the same suppliers, with standardized prices. Employees are part of outsourced companies, all with the same standard salary, and they participate in the same training types; the production area structure is also similar. Therefore, the consumption of electricity and the average gas is similar. Due to schools’ similarities, they do not show significant changes among different menus. Thus, almost all items of this dimension had the same severity rating in all schools. However, each item’s score varied from one school to another due to the difference in the proportion of wasted food produced/food produced.

Only one item varied among the evaluated days in the economic dimension: the cost of raw materials, which changed according to the daily offered menu. However, when comparing schools, the SF was constant and similar. The menus offered in different schools were similar.

Although employees’ training is standardized and offered to all by the same outsourced company, structural differences and availability of utensils and equipment were noticed within the schools’ sample. It is important to note that, although the eco-inefficiency formula indirectly indicates structural factors, during the data collection for the application of the EIy, the structure data were also collected from the completion of a checklist available in the Guide for Good Practices in School Feeding [66]. Structural issues of the place of production and food distribution can be associated with the generation of waste.

A study carried out in the State of Paraíba evaluating the structure and adequacy of the National School Meals Program (PNAE) in the region pointed out several structural and procedural flaws in the program’s execution, such as deficiencies in the structure of the kitchens and cafeterias (which sometimes did not even exist), and flaws in nutritional education activities, which are part of the PNAE policy [67]. Corroborating the findings of Pedraza (2017), in the Pilot Study conducted in Brasilia, the reality of the structure of schools’ FS did not much favor the appropriated consumption of food. No school monitored during the pilot study had a dedicated space for feeding students. Thus, even if indirectly, applying the EIy formula in FS allowed us to perceive some limitations in managing meals’ production and distribution.

Due to the nature of the convenience sample used in the pilot study and the reduced number of FS (n = 4) allowing the evaluation of 20 menus, the results are not representative of all FS in public schools in FD. We also emphasize that items of all dimensions often have correlations. Even if they are classified in a specific dimension, observations must always be made considering all aspects so that no change made is harmful to another dimension. Thus, EIy must be continuously applied to assess changes and conduct, since all items correlate, and then modify them as needed.

The present study has limitations regarding the inability of quantifying inedible parts of the food waste, but the possibility of including this item in the formula should be evaluated in the future. Another limitation is developing a formula in which data from the local reality (Brazil) is used as a reference. Nevertheless, the detailed description of the method, adaptations to other places’ realities, and local references allow its national and international application. Thus, for the EIy formula to be used, it is necessary to stick to the defined items and determine SF’s appropriate scales following local references, preferably defined at three levels. Therefore, this study demonstrated that the application of EIy is an easy and practical way to identify the cost of food waste. It indicates the main factors influencing waste in evaluated FS or menus and stands out as an important way to assess the impacts generated by food waste.

Finally, this study is an initial part of a larger project that foresees applying the formula in other FS. It will make it possible to diagnose the region’s reality and propose several solutions that reduce food waste in this segment.

## 4. Conclusions

The developed eco-inefficiency formula is an indicator for restaurants that their processes present flaws that directly or indirectly impact food waste’s social, environmental, and economic aspects. The results allowed us to obtain a standardized impact score generated exclusively by food waste. The pilot study confirmed the feasibility of applying the EIy formula and each items’ evaluation allowed us to understand EIy as an indicator of the FS failures that impact food waste. The pilot study indicated that the environmental dimension demands the most attention in the FS evaluated, requiring strategies that reduce the waste’s impact.

The 13 constructs listed to score eco-inefficiency in food services, divided between the three dimensions of sustainability, should be evaluated by those responsible for food services regarding the need for corrective actions directed at specific points. EIy allows continuous monitoring of services, evaluation of plans and procedures related to waste reduction and helps to reduce the impact of food waste in all dimensions of sustainability, favoring food service and society in general.

Although the formula highlights the critical points for intervention, its application must be constant and careful, with a holistic view of the situation. The formula also allows comparison between menus or even between establishments, identifying behaviors and actions that favor or do not favor environmental conservation, with reduced costs and nutritional benefits for consumers. In this way, it allows food services to be continuously monitored and to evaluate their plans and procedures related to waste reduction.

The current study considered the variables perceived at the moment, but it must be updated continuously since this is an extremely adaptable formula to each food service’s reality. New studies are necessary to validate the formula’s application in other FS types. The new formula can also be used in other countries, with appropriate adjustments being made to legislation or local reference classifications.

## Figures and Tables

**Table 1:** Dimensions and parameters for the calculation and classification of eco-inefficiency in restaurants.

Economic dimension				
Item	Definition	Reference	Parameter evaluated in the restaurant	Classification
Cost of raw material in food waste (%)	The proportion of the cost of wasted raw material.	Best considered values are below 3% [25].	Value of acquisition of each ingredient used.	0 to 3% 3.01% to 10% Above 10%
Gas consumption	Volume of the gas cylinder and frequency of its change.	Fare range for industrial and commercial clients—conventional consumption made available by a Brazilian company [26]. Consumption ranges are considered low, medium, or high by the local company.	Information provided by the evaluated establishment (m^3^ LPG/day).	0 to 5.1 m^3^/day 5.1 to 100 m^3^/day Above de 101 m^3^/day
Energy	Energy consumption during food production.	Values provided by the electricity company where the restaurant is located [18].	Record of the connected devices, daily operating time for each device, and the average consumption declared by the manufacturer.	USD 0.15/kWh USD 0.17/kWh USD 0.18/kWh
Salary of food handlers	Food handlers’ mean wage per day.	Distributed in low, medium, and high according to the last classification made by IBGE [27].	The daily mean wage of a food handler wasted.	Below USD 35.39 From USD 35.4 to USD 147.46 Above USD 147.47
**Environmental Dimension**				
Water footprint (WF)	Water volume used directly or indirectly in the production of food for Ely formula, the WF of the wasted food.	Animal ingredients [20]. Vegetal ingredients [21,28].	Cutoff points are defined by terciles (pilot study).	Low: ∑ WF < 10.000 Medium: 10.000 ≤ ∑ WF < 30.000 High: ∑ WF ≥ 30,000
Cleaning material	Proper use of the product according to the manufacturer’s recommendation (dilution, exposure time).	Product manufacturer.	Proper use during food production	Adequate Inadequate
Food production waste	Food Shavings/gross weight × 100	Considered best values below 3% [25] and acceptable up to a maximum of 10% of what was produced [29].	The amount of food discarded during production (food shavings).	0 to 3% 3.01% to 10% Above 10%
Amount of rest-intake	Amount (in kg) of food discarded after consumption in the plates of consumers.	For the amount of rest-intake and distribution leftover, we used the same categorization as food production waste.	Direct weighing of rest-intake and distribution leftover.	0 to 3% 3.01% to 10% Above 10%
Amount of distribution leftover	Amount (in kg) of leftover food after distribution that was not in consumers’ plates.			0 to 3% 3.01% to 10% Above 10%
**Social dimension**				
The energy density (ED) of rest- intake	ED = Kcal of rest-intake/Kg of rest-intake	Defined as a low, medium, and high ED, defined by the Centers for Disease Control and Prevention (2005) [23].	Calculation of ED of the menu served by the restaurant.	Low: 0.00 to 1.50 kcal/g Medium: 1.51 to 4.00 kcal/g High: 4.01 to 9.00 kcal/g
The energy density (ED) of distribution leftover	ED = Kcal of distribution leftover/Kg of distribution leftover	Defined as a low, medium, and high ED, defined by the Centers for Disease Control and Prevention (2005) [23].	Calculation of ED of the menu served by the restaurant.	Low: 0.00 to 1.50 kcal/g Medium: 1.51 to 4.00 kcal/g High: 4.01 to 9.00 kcal/g
Organic food use	Organic foods or other types of sustainable production that favor the health of consumers and producers.	It is considered a sustainable restaurant with more than 50% of fruits and vegetables with an organic seal [24].	Identify the percentage of foods on the menu that have organic certification or sustainable production.	Up to 50% of fruits and vegetables from sustainable production Below 50% of sustainable production fruits and vegetables
Food donation	Considered when the donation of food is allowed under adequate conditions of human consumption, it can be donated to people in vulnerable situations.	Law 14.016, of 23 June 2020, which provides for combating food waste and the donation of surplus food for human consumption [30].	Disposal of food surpluses under conditions of human consumption (leftover food)	Excess food in suitable conditions is donated for human consumption Distribution leftovers are destined for composting, animal feed, or another sustainable alternative All food is disposed of in the conventional garbage collection system.

**Table 2:** Eco-inefficiency average, standard deviation (±SD), and percentage of the score (%) of the environmental, social, and economic impacts of each school’s waste in a five-day menu evaluation in the pilot study.

Dimension	ITEM	School 1			School 2			School 3			School 4		
		Score	Total by Dimension	%	Score	Total by Dimension	%	Score	Total by Dimension	%	Score	Total by Dimension	%
Economic	Raw material cost	0.77 ± 0.05	2.48	62	0.80 ± 0.07	2.53	63.3	0.68 ± 0.09	1.91	47.8	0.73 ± 0.08	2.24	56
	Electricity	0.77 ± 0.09			0.71 ± 0.09			0.57 ± 0.11			0.73 ± 0.07		
	Liquefied petroleum gas	0.47 ± 0.09			0.51 ± 0.13			0.33 ± 0.12			0.39 ± 0.12		
	Handler’s salary	0.47 ± 0.09			0.51 ± 0.13			0.33 ± 0.12			0.39 ± 0.12		
Environmental	Water footprint	0.68 ± 0.09	3.67	73.4	0.71 ± 0.12	3.73	74.6	0.57 ± 0.25	3.18	63.9	0.39 ± 0.20	3.20	64
	Cleaning material	0.77 ± 0.05			0.80 ± 0.07			0.68 ± 0.09			0.68 ± 0.09		
	Food shavings	0.68 ± 0.19			0.71 ± 0.04			0.57 ± 0.21			0.73 ± 0.25		
	%Rest-intake	0.77 ± 0.05			0.80 ± 0.07			0.68 ± 0.09			0.62 ± 0.13		
	%Distribution leftover	0.77 ± 0.05			0.71 ± 0.13			0.68 ± 0.15			0.73 ± 0.14		
Social	Energy density of rest-intake	0.47 ± 0.09	2.48	62	0.51 ± 0.13	2.62	65.5	0.33 ± 0.12	2.02	50.5	0.39 ± 0.12	2.24	56
	Energy density of distribution leftover	0.47 ± 0.09			0.51 ± 0.13			0.33 ± 0.12			0.39 ± 0.12		
	Organic food	0.77 ± 0.05			0.80 ± 0.07			0.68 ± 0.09			0.73 ± 0.08		
	Food donation	0.77 ± 0.05			0.80 ± 0.07			0.68 ± 0.09			0.73 ± 0.08		

## Data Availability

The study did not report any data.

## Supplementary Materials

The following are available online at https://www.mdpi.com/article/10.3390/foods10061369/s1, Table S1. Week menu per day of each school analyzes in the study of eco-inefficiency formula; Table S2. Model of Technical Preparation Files Form; Table S3. “Wasted food/Meal produced” by day of each school analyzed in the pilot study of eco-inefficiency formula; Table S4. Average of the values of each variable collected and their respective classification according to the Severity Factor (SF) for schools analyzed in the pilot study of eco-inefficiency formula.
