# Supplementary material for: Eco-Inefficiency Formula: A Method to Verify the Cost of the Economic, Environmental, and Social Impact of Waste in Food Services
Source: Foods. 2021 Jun 13;10(6):1369. doi: 10.3390/foods10061369 (PMC8231818; doi:10.3390/foods10061369)
Supplement: Supplementary file 1 [file foods-10-01369-s001.zip › foods-1252524-supplementary.pdf]

Table S1. Week menu per day of each school analyzes in the study of eco-inefficiency formula

WEEK MENU					
School Code	Monday	Tuesday	Wednesday	Thursday	Friday
1	Rice with pork	Noodle soup with chicken, pumpkin, and tomato	Chicken, crumbs with cabbage and apple	Bread with chicken breast and pineapple juice	Mapará (fish) fillet with potatoes, green peppers and rice and grated beet salad with tomato
2	Rice with beans and pork palette	Fish with mush, white rice, and cucumber with carrot salad	Pasta with chicken and melon	Chicken with cucumber and carrot salad	Shortbread with banana smoothie
3	Rice with beans and pork palette	Fish with flour and own sauce, white rice, and tangerine	Shortbread and dairy compound	Pasta with chicken breast	Chicken with boiled sweet potatoes
4	Cornstarch biscuit with dairy compound	Pork palette with carrot, rice, beet with broccoli and tangerine	Bread with chicken and pineapple juice	Noodle soup with pork and carrots, cabbage and kale	Banana smoothie with cornstarch biscuit

Table S2. Model of Technical Preparation Files Form

<table>
<tr><td colspan="7">Technical Preparation Files</td></tr>
<tr><td colspan="2">Restaurant Identification:
____
_</td><td colspan="5">Date: ___/____/______ Researcher:
__________________________</td></tr>
<tr><td colspan="3">Preparation:</td><td colspan="4">Number of diners:</td></tr>
<tr><td>Yield:
Kg</td><td colspan="2">Production leftover:
Kg</td><td>Rest-intake:
Kg</td><td colspan="2">Portion:
(g)</td><td>Homemade measure:</td></tr>
<tr><td colspan="3">Ingredients</td><td colspan="2">GW (g)</td><td colspan="2">NW (g)</td></tr>
<tr><td colspan="3"></td><td colspan="2"></td><td colspan="2"></td></tr>
<tr><td colspan="3"></td><td colspan="2"></td><td colspan="2"></td></tr>
<tr><td colspan="3"></td><td colspan="2"></td><td colspan="2"></td></tr>
<tr><td colspan="3"></td><td colspan="2"></td><td colspan="2"></td></tr>
<tr><td colspan="3"></td><td colspan="2"></td><td colspan="2"></td></tr>
<tr><td colspan="3"></td><td colspan="2"></td><td colspan="2"></td></tr>
<tr><td colspan="3"></td><td colspan="2"></td><td colspan="2"></td></tr>
<tr><td colspan="3"></td><td colspan="2"></td><td colspan="2"></td></tr>
<tr><td colspan="7">PREPARATION MODE</td></tr>
<tr><td colspan="7"></td></tr>
</table>

Table S3. “Wasted food/Meal produced” by day of each school analyzed in the pilot study of eco-inefficiency formula

		Day				
		1	2	3	4	5
School	1	0.38	0.54	0.37	0.53	0.52
	2	0.68	0.44	0.55	0.34	0.56
	3	0.43	0.36	0.30	0.15	0.43
	4	0.20	0.38	0.44	0.51	0.42

Table S4. Average of the values of each variable collected and their respective classification according to the Severity Factor (SF) for schools analyzed in the pilot study of eco-inefficiency formula

		School 1		School 2		School 3		School 4	
Dimension	ITEM	Data collected	SF	Data collected	SF	Data collected	SF	Data collected	SF
Economic	Raw Material Cost	46.77	3	51.28	3	33.11	3	39.21	3
	Electricity	0.39	3	0.39	3	0.29	3	0.84	3
	LPG[1]	0.60	1	0.60	1	0.60	1	0.60	1
	Handler's salary	16.15	1	17.7	1	11.45	1	13.55	1
Ambiental	Water Footprint	12954.93	2	32027.04	2	14502.11	2	9491.31	1
	Cleaning Material	Incorrect	3	Incorrect	3	Incorrect	3	Incorrect	3
	Food shavings	8.40	2	18.23	3	3.48	2	15.50	3
	%RI[2]	20.71	3	33.95	3	14.00	3	7.72	2
	%DL[3]	18.37	3	10.80	3	15.89	3	14.51	3
Social	ED RI	0.78	1	0.85	1	0.94	1	0.96	1
	ED DL	0.78	1	1.34	1	1.49	1	1.52	1
	Organic food	0	3	0	3	0	3	0	3
	Food destination	-	3	-	3	-	3	-	3

[1] Liquefied petroleum gas
[2] Rest-intake
[3] Distribution leftover
